# Interface-sensitive imaging by an image reconstruction aided X-ray reflectivity technique^1^

**DOI:** 10.1107/S160057671700509X

**Published:** 2017-05-25

**Authors:** Jinxing Jiang, Keiichi Hirano, Kenji Sakurai

**Affiliations:** a University of Tsukuba, 1-1-1 Tennodai, Tsukuba, Ibaraki 305-0006, Japan; b National Institute for Materials Science, 1-2-1 Sengen, Tsukuba, Ibaraki 305-0047, Japan; c Photon Factory, High Energy Accelerator Research Organization, KEK, 1-1 Oho, Tsukuba, Ibaraki 305-0087, Japan

**Keywords:** surfaces and interfaces, micro-imaging, X-ray reflectivity, image reconstruction, visualization

## Abstract

Recently, the authors have succeeded in realizing X-ray reflectivity imaging of heterogeneous ultrathin films at specific wavevector transfers by applying a wide parallel beam and an area detector. By combining in-plane angle and grazing-incidence angle scans, it is possible to reconstruct a series of interface-sensitive X-ray reflectivity images at different grazing-incidence angles (proportional to wavevector transfers). The physical meaning of a reconstructed X-ray reflectivity image at a specific wavevector transfer is the two-dimensional reflectivity distribution of the sample. In this manner, it is possible to retrieve the micro-X-ray reflectivity (where the pixel size is on the microscale) profiles at different local positions on the sample.

## Abstract 2

This article describes interface-sensitive imaging of heterogeneous thin films by an image reconstruction aided X-ray reflectivity technique with an 8 mm-wide parallel beam; the possibility of extracting micro-X-ray reflectivity profiles from the same data collection is discussed.

## 1. Introduction   

The significance of interfaces cannot be overstated, with their ubiquity from the hardware of the information age to the processes of life (Allara, 2005 ▸). The unique molecular and atomic features of the interfaces between materials often control many functions of both naturally occurring and synthetic materials (Chandler, 2005 ▸; Yin & Alivisatos, 2005 ▸). Interfaces play vital roles in the functions of materials as diverse as the rate of an electrochemical process, the adhesive strength and conductivity of a thin metal-film coating, the compatibility of a biological implant, the efficiency of a semiconductor transistor, and the corrosion of a structural metal induced by its working environment.

X-ray reflectivity is a powerful technique for studying buried interfaces in ultrathin films in a non-destructive manner (Daillant & Gibaud, 1999 ▸; Holý *et al.*, 1999 ▸; Parratt, 1954 ▸; Sinha *et al.*, 1988 ▸; Holý & Baumbach, 1994 ▸; Stoev & Sakurai, 1999 ▸). However, routine X-ray reflectivity assumes that the sample to be measured is in-plane homogeneous, which is not the case in many structures. As such, imaging capabilities are essential for modern interface characterization, yet only a few X-ray techniques (Fenter *et al.*, 2006 ▸; Roy *et al.*, 2011 ▸; Sun *et al.*, 2012 ▸) have been developed for imaging interfaces in the past decade.

Recently, the authors have successfully developed a complementary novel X-ray reflectivity imaging (XRI) technique employing a wide monochromatic synchrotron beam (Jiang *et al.*, 2016 ▸) and an area detector. This technique (Innis-Samson *et al.*, 2011 ▸, 2012 ▸; Jiang & Sakurai, 2016 ▸) is based on X-ray reflectivity and an image reconstruction scheme that is mathematically similar to computed tomography (Kak & Slaney, 1999 ▸; Natterer, 2001 ▸; Herman, 2009 ▸). The physical meaning of a reconstructed X-ray reflectivity image at a specific wavevector transfer is the two-dimensional reflectivity distribution of the sample. The present work extends the technique to obtain more information on the samples by collecting a series of X-ray reflectivity images at different wavevector transfers. It is possible to retrieve many X-ray reflectivity profiles at microscale regions covering the full area of a sample (size: 8 × 8 mm).

## 2. Experimental   

### 2.1. Model sample preparation   

**Figure 1:**
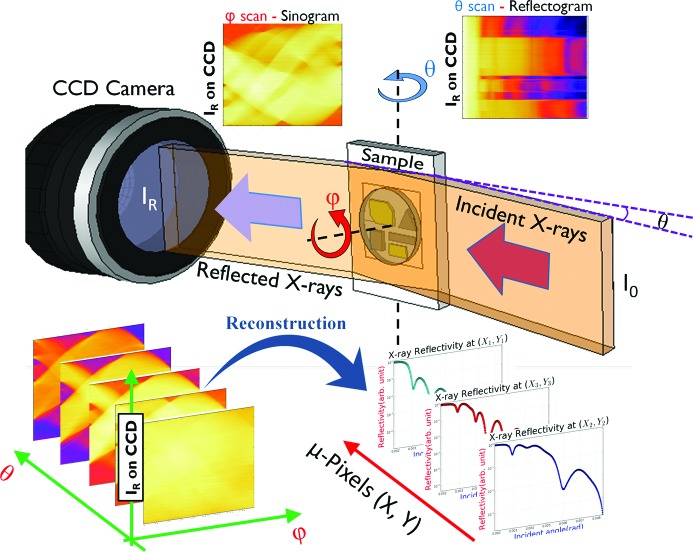
Conceptual schematic of the interface-sensitive imaging technique by image reconstruction aided X-ray reflectivity. A monochromatic wide X-ray beam irradiates the full sample at a grazing-incidence angle θ and the reflected X-ray beam at the equivalent exit angle θ is recorded as an approximate one-dimensional profile by an X-ray CCD camera. The sample is rotated in-plane and many such one-dimensional profiles are recorded at different in-plane angles φ (usually plotted as a sinogram). By combining these scans with the grazing-incidence angle θ scan, many sinograms at different θ are collected as the raw data. The full µXR profiles from different sample positions are derived from the collection of the whole data set by a reconstruction process.

The sample measured was a heterogeneous patterned ultrathin film sample, as schematically shown in the centre of Fig. 1 ▸. The yellow polygons correspond to gold (Au) thin films and the brown polygons to nickel (Ni) thin films, and the transparent flat cylinder denotes the uniform titanium (Ti) covering layer. The sample composed of heterogeneous layers was fabricated with an Eiko DID-5A magnetron sputtering system on a pre-cleaned silicon substrate (20 × 15 × 2 mm). Under the top uniform Ti layer the heterogeneous layer is composed of two groups of thin films: (i) Au thin films including the top-left polygon and bottom-right rectangle with different thicknesses; (ii) Ni thin films consisting of the bottom-left thick rectangle, top-right triangle and centre-right thin bar (see the schematic of the sample in Fig. 1 ▸). The model sample was constructed as follows: the silicon substrate was set into the sputter chamber and covered by a series of masks made of Kapton film. The masks were pre-cut with the different designed patterns. The chamber of the sputtering machine was pre-evacuated to <0.1 Pa and filled with argon (Ar) gas. High voltage was applied, ionizing Ar atoms to sputter off the original Au material, and first the gold rectangular film was deposited onto the bottom right of the substrate. The sputtering conditions were as follows: Ar pressure 2 Pa; ion current 50 mA; sputtering time 30 s. The mask was then replaced by that of a different pattern. The sputtering chamber was again pre-evacuated to <0.1 Pa and filled with Ar gas. High voltage was then applied and the sputtering conditions for the second Au pattern were as follows: Ar pressure 2 Pa; ion current 50 mA; sputtering time 60 s. The Au foil target was replaced by an Ni foil target. The Ni patterns (triangle, rectangle and bar) were then deposited one by one. The sputtering conditions to prepare different Ni patterns with different thicknesses were as follows: Ar pressure 2 Pa; ion current 260 mA; sputtering time 10 s (for the centre-right thin bar), 20 s (for the top-right triangle) and 30 s (for the bottom-left thick rectangle). The Ni foil target was then replaced by a Ti foil target, and a mask with a circular hole (8 mm diameter) was set to limit the deposited area. The sputtering chamber was again pre-evacuated to <0.1 Pa and filled with Ar gas. The sputtering conditions for the Ti layer were as follows: Ar pressure 2.0 Pa; ion current 200 mA; sputtering time 80 s. The Ti layer accordingly covers the Au and Ni patterns such that the overall thickness of the heterogeneous sample is uniform and the Au and Ni regions are buried, separated layers.

### 2.2. XRI technique   

The experiments of interface-sensitive imaging by X-ray reflectivity were carried out on beamline 14B, the Photon Factory, Tsukuba, Japan. The new imaging approach is an extension of the recently developed XRI technique. By combining XRI and the ordinary X-ray reflectivity (XR) θ/2θ scan, one can realize interface-sensitive imaging, as schematically shown in Fig. 1 ▸. (In the figure the in-plane angle φ = 0° and grazing-incidence angle θ = 2 mrad are shown.)

The experimental setup is the same as that of XRI (Jiang *et al.*, 2016 ▸). The synchrotron radiation from the vertical wiggler was monochromated to 16 keV (around the peak position of the spectrum; Ando *et al.*, 1986 ▸) by a fixed-exit double-crystal Si(111) monochromator, with an energy resolution of ∼10^−4^. The monochromatic X-rays were collimated by several slits to form a parallel beam (vertical angular divergence 0.02 mrad). The primary collimating four-dimensional slit was set at the furthest upstream side of the experiment hutch, which was 22.5 m away from the wiggler source, to collimate the beam to 1 mm (horizontal, H) × 8 mm (vertical, V). The X-ray intensity was monitored throughout the experiment by an ionization chamber (IC) set 0.45 m behind the four-dimensional slit. In front of the entrance window of the IC, a fixed-width (100 µm, H) slit was attached to further cut the horizontal width of the beam; thus, the final incident-beam size was 0.10 mm (H) × 8 mm (V) at the IC position. The sample stage, which was set at 0.45 m downstream from the IC, is based on a high-precision θ/2θ goniometer with an accuracy of 0.001°. A rotational motor is vertically attached to an L-shaped stand fixed on the goniometer to realize in-plane φ rotation. The samples were vertically mounted using a sample holder that employs a small pump to attach the substrate from the backside. The sample holder was equipped with two manual tilt stages to adjust the sample surface to be perpendicular to the in-plane rotational axis. The parallel beam illuminated around 10 mm [H, the footprint length of the X-rays is always long enough to cover the silicon substrate size (10 mm)] × 8 mm (V) of the sample surface at grazing-incidence geometry. The reflected X-rays were recorded by an X-ray CCD camera (pixel size 6.45 µm) set 0.30 m on the downstream side of the sample as a one-dimensional projection image, where the imaging conditions are in the near-field regime. For the in-plane angle φ scan, the sample was rotated in-plane in angular steps of Δφ = 2° (*N* = 90 projections) up to 180°; reflection projections were collected at each angle and plotted as a sinogram. For the grazing-incidence angle θ scan, the sample was tilted in grazing-angle increments of Δθ = 0.004°; reflection projections were collected as a function of θ (θ_min_ = 0.1080°, θ_max_ = 0.4800°) and plotted as a reflectogram. The corresponding *Q_z_* range is 0.031–0.137 Å^−1^, in which the specular reflection is very dominant and the influence of diffuse scattering is almost negligible. In the horizontal direction, the footprint length along the X-ray forward direction is *L* = *d*/sinθ. The smallest footprint on the sample is *L*_min_ = *d*/sinθ_max_ = 12 mm, which is still larger than the size of the sample. By combining the φ scan (XRI) and θ scan (XR), it is possible to reconstruct the micro-X-ray reflectivity (µXR) profiles at different local positions of the sample, where the in-plane spatial resolution of the µXR is limited by the pixel size of the reconstructed XRI images.

## 3. Results and discussion   

### 3.1. Raw data reduction   

The raw data were reflection projections recorded by the CCD camera and stored in many TIF (tagged image format) images with 16 bit dynamic range. It is necessary to mention that the X-rays’ footprints on the CCD camera are not perfectly one-dimensional projections but narrow rectangles as the incident X-rays have a horizontal width of 100 µm. In order to efficiently handle many TIF images, several Python open-source libraries designed for scientific computing such as *NumPy* (Oliphant, 2007 ▸), *Matplotlib* (Hunter, 2007 ▸) and *Tkinter* (Lundh, 1999 ▸; Shipman, 2010 ▸) have been employed. For the data reduction and processing, some additional Python codes have been prepared to read each TIF file, specify the area of interest and integrate the reflection rectangles into one-dimensional projections with batch mode compatibility. The CCD dark count background is subtracted for each image and then the data are normalized to counting rate by considering different measuring times for low and high grazing-incidence angles.

### 3.2. Data collection   

In the measurements, one-dimensional X-ray reflection projections were collected systematically at different grazing angles (θ scan) and in-plane angles (φ scan). The reduced data are composed of many one-dimensional X-ray reflection projections as a function of grazing angle θ and in-plane angle φ. They can be grouped as either (*a*) different sinograms at different grazing angles or (*b*) different reflectograms at different in-plane angles. The former grouping method is equivalent to many XRI measurements at different grazing angles, while the latter grouping method corresponds to many XR measurements at different in-plane angles. In order to retrieve µXR at all the in-plane locations of the sample, both φ scans and θ scans are required. The order can be chosen freely depending on experimental convenience.

#### 3.2.1. Sinograms at different wavevector transfers *Q_z_*   

**Figure 2:**
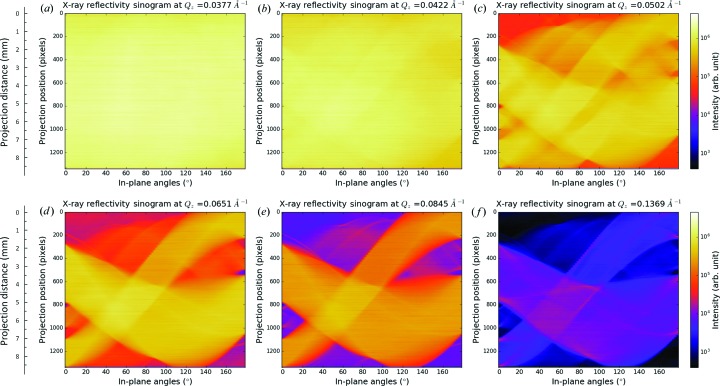
Selected XR sinograms of the sample plotted as a function of in-plane angle φ at wavevector transfers of (*a*) *Q_z_* = 0.0377 Å^−1^, (*b*) *Q_z_* = 0.0422 Å^−1^, (*c*) *Q_z_* = 0.0502 Å^−1^, (*d*) *Q_z_* = 0.0651 Å^−1^, (*e*) *Q_z_* = 0.0845 Å^−1^ and (*f*) *Q_z_* = 0.1369 Å^−1^, where the data are plotted on the same logarithmic colour scale. The scanning step for the measurement is Δφ = 2°.

In the same fashion as XRI, the experimental data were stored as a collection of sinograms at different grazing-incidence angles θ, namely at corresponding wavevector transfers *Q_z_*. θ is the grazing-incidence angle and is related to the wavevector transfer *Q_z_* by (Als-Nielsen & McMorrow, 2011 ▸) 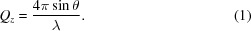
Fig. 2 ▸ gives some selected X-ray reflectivity sinograms of the sample at specific incidence angles with wavevector transfers of (*a*) *Q_z_* = 0.0377 Å^−1^, (*b*) *Q_z_* = 0.0422 Å^−1^, (*c*) *Q_z_* = 0.0502 Å^−1^, (*d*) *Q_z_* = 0.0651 Å^−1^, (*e*) *Q_z_* = 0.0845 Å^−1^ and (*f*) *Q_z_* = 0.1369 Å^−1^. The full collection of sinograms at different wavevector transfers can be found in video 1 in the supporting information. Mathematically, a reflection projection at a specific wavevector transfer is the integral reflection intensity profile along the X-ray forward direction according to the Radon transform (Herman, 2009 ▸): 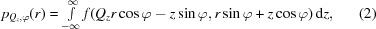
where *Q*_*z*_ is the chosen wavevector transfer, φ is the in-plane angle, *r* is the projection position (the experimental pixel number on the CCD camera), *z* is the X-ray forward direction, *f*(*x*, *y*) is the reflection intensity at the sample position (*x*, *y*), and *p*_*Q_z_*__,φ_(*r*) is the one-dimensional integrated reflection projection profile at the specific *Q*_*z*_ and the in-plane angle φ. In each panel of Fig. 2 ▸, the features (if any) experience a half rotation; thus the integrated reflection projection forms a half period of a sine wave. The X-ray’s penetration depth in the sample is tuned by the wavevector transfer *Q_z_*. At small wavevector transfer *Q_z_* = 0.0377 Å^−1^, the X-rays are totally reflected by the Ti surface, thus producing a uniform sinogram. In panel (*b*) where *Q_z_* = 0.0422 Å^−1^, an indistinct feature is immersed in the uniform background. However, when *Q_z_* = 0.0502 Å^−1^, a strong contrast is achieved and the pattern below the Ti is visible as *Q_z_* is beyond the critical wavevector transfer of Ti [*Q*_c_(Ti)]. In panel (*d*) where *Q_z_* = 0.0651 Å^−1^, variation begins to exist between different features, as Ni patterns are weakly reflected at this specific *Q*_*z*_, which is larger than Ni’s critical wavevector transfer *Q*_c_(Ni). In panels (*e*) *Q_z_* = 0.0845 Å^−1^ and (*f*) *Q_z_* = 0.1369 Å^−1^, both of the wavevector transfers are larger than Au’s critical wavevector transfer *Q*_c_(Au), and the change of contrast in the sinograms is due to the detailed characteristics inside the gold thin films and the nickel thin films. In the above *Q_z_* range, the contribution of the diffuse scattering, which may smear the contrast in the reflection profile, was negligible.

#### 3.2.2. Reflectograms at different in-plane angles φ   

**Figure 3:**
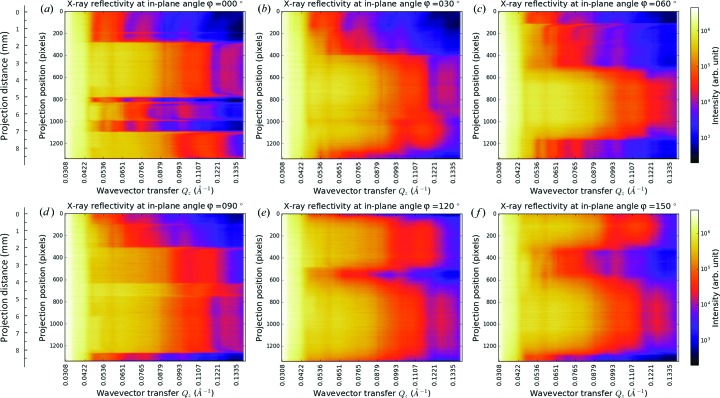
Selected X-ray reflectograms of the sample plotted as a function of wavevector transfer *Q_z_* at specific in-plane angles: (*a*) φ = 0°, (*b*) φ = 30°, (*c*) φ = 60°, (*d*) φ = 90°, (*e*) φ = 120° and (*f*) φ = 150°, where the data are plotted on the same logarithmic colour scale. The scanning step for the measurement is Δ*Q_z_* = 0.00114 Å^−1^.

The collected data can be categorized in another manner: a group of reflectograms at different in-plane angles. A reflectogram is composed of reflection projections at a series of wavevector transfers collected by a θ scan. The full collection of reflectograms at different in-plane angles is provided in video 2 in the supporting information. Six selected reflectograms at characteristic in-plane angles are shown in Fig. 3 ▸. In every panel, a sharp intensity drop at *Q_z_* = 0.042 Å^−1^ is observed, which corresponds to the critical wavevector transfer *Q*_c_ of Ti. Another two intensity drops are apparent at around *Q_z_* = 0.050 Å^−1^ and *Q_z_* = 0.080 Å^−1^, corresponding to *Q*_c_ of Ni and Au, respectively. More careful inspection of the critical wavevector transfers *Q*_c_ from the experimental data and comparison with theoretical values will be done in §3.4. A reflectogram is physically a collection of one-dimensional reflection projections integrating along a specific observation direction at a series of wavevector transfers. Consider panel (*a*) at φ = 0°: (i) In the position range of [0–280], where the sample is composed of a uniform Ti layer, four equal-period interference fringes are observed (the same feature is seen throughout the whole reflectogram, and also in the range of [1000–1080]). (ii) In the position ranges of [280–780] and [1080–1338], there are higher reflectivity intensities in the whole *Q_z_* range than in other ranges. Such areas are a mixture of Au and Ni patterns, and the contribution of reflection intensities from Ni is weak beyond the critical wavevector transfer of Ni [*Q*_c_(Ni) = 0.050 Å^−1^]. The contribution of Ni patterns is still visible in the position ranges of [500–780] and [1240–1338] where there exist low intensities in the range of *Q_z_* = 0.042–0.050 Å^−1^ corresponding to a lack of Ni patterns (see the schematic of the sample in Fig. 1 ▸ for comparison). In that range X-rays are not only totally reflected by Au but also completely reflected by Ni. The reflection intensity profiles in the position ranges of [280–780] and [1080–1338] are also different, implying different structures of the Au and Ni patterns. (iii) In the position range of [820–1000], the reflection intensity profile is not the same for each position, which implies different thicknesses at local positions of the thin film. (iv) At the position around [200], there exists a brighter reflection intensity profile, which corresponds to the leakage of Au from the mask in the sputtering process (as will be discussed in §3.3).

In panel (*b*) at φ = 30° and in panel (*c*) at φ = 60°, the reflectograms have different characteristics compared with that in panel (*a*): the reflection intensity profiles of the two bright patterns are closer and the patterns overlap with each other and form a higher-intensity region in the position range of [520–1180] at φ = 60°. In the position range of [120–260], there appears a new pattern that corresponds to the Ni triangle on the sample. When the sample rotates to the in-plane angle φ = 90° in panel (*d*), the separation and the other side of the Au polygon and rectangle of differing length become apparent. In panels (*e*) and (*f*), the reflectograms give further different descriptions of the sample at specific in-plane angles. How the reflectogram changes with in-plane angle is demonstrated in video 2 in the supporting information.

### 3.3. Reconstructed X-ray reflectivity images   

As it is possible to measure the reflection intensity of every pixel in an XRI image (Jiang *et al.*, 2016 ▸; Jiang & Sakurai, 2016 ▸) from a sinogram at a specific wavevector transfer *Q_z_* and a sufficient number of sinograms at different *Q_z_* values, the µXR profile at every pixel is easily plotted by extracting reflection intensities from a series of XRI images. In order to achieve a quantitative XR profile, a stable image reconstruction scheme shall be adopted. In the present work, after transferring the Radon transfers to an algebraic linear system (Kak & Slaney, 1999 ▸; Natterer, 2001 ▸; Herman, 2009 ▸), the pseudoinverse algorithm (Strang & Borre, 1997 ▸; Hansen, 1997 ▸, 2010 ▸) has been applied (specifically, the truncated singular value decomposition method) to reconstruct the XRI images. Because it is a direct discrete method and a convenient way to apply different regularization methods to different sample cases, the algebraic approach has been employed to quantitatively reconstruct the XRI images.

**Figure 4:**
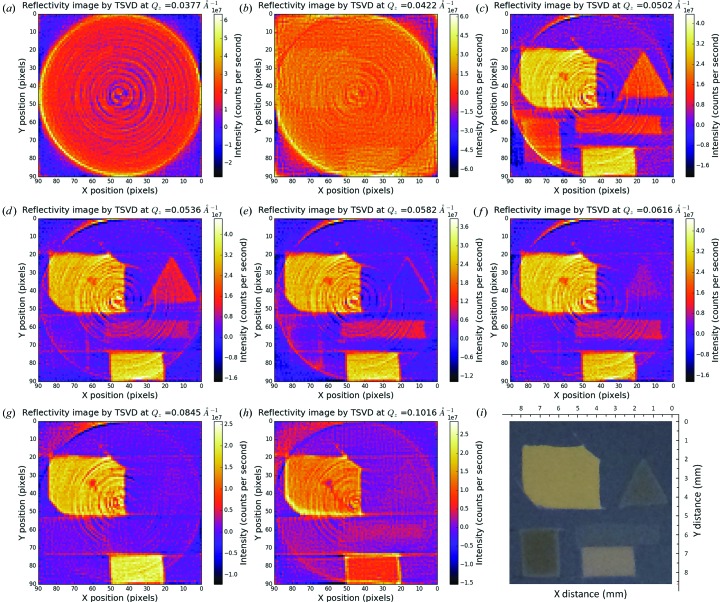
Selected reconstructed XR images [by the truncated singular value decomposition (TSVD) method] of the sample at wavevector transfers of (*a*) *Q_z_* = 0.0377 Å^−1^, (*b*) *Q_z_* = 0.0422 Å^−1^, (*c*) *Q_z_* = 0.0502 Å^−1^, (*d*) *Q_z_* = 0.0536 Å^−1^, (*e*) *Q_z_* = 0.0582 Å^−1^, (*f*) *Q_z_* = 0.0616 Å^−1^, (*g*) *Q_z_* = 0.0845 Å^−1^ and (*h*) *Q_z_* = 0.1016 Å^−1^, where the data are plotted on linear colour scales. The number of projections for image reconstruction: 90 views. (*i*) An optical image of the patterned sample before the Ti layer was deposited. The image was trimmed to have the same scale as the reconstructed images.

In the experiment, reflection projections at 90 in-plane angles per wavevector transfer have been measured. In order to avoid the rank-deficient problem of the algebraic system (Aster *et al.*, 2011 ▸), the 1338 pixels (measured one-dimensional reflection projection length: 6.45 µm × 1338 = 8.6 mm) are equally binned into 90 pixels (pixel length: 96 µm, 96 µm × 90 = 8.6 mm), yet the spatial resolution suffers from the binning process. The pixel size and/or pixel separation distance is α = 96 µm. The sample-to-detector distance is *R* = 300 mm and then *R* ≪ α^2^/λ = 120 m is obtained. Thereby the imaging conditions are in the near-field regime, as stated in §2.2. In order to achieve higher-resolution µXR, a smaller in-plane angle step scan is necessary. Fig. 4 ▸ presents selected reconstructed XR images of the sample at various wavevector transfers. No image correction has been attempted to remove ring artefacts (Raven, 1998 ▸; Sijbers & Postnov, 2004 ▸) caused by the inhomogeneity of the detector response. Panel (*i*) is the optical image of the sample taken before the Ti layer was deposited.

Panel (*a*) shows a uniform image, irrespective of the existence of ring artifacts. The uniformity of this image just matches the uniform surface of the Ti layer, whose *Q*_c_(Ti) = 0.042–0.0377 Å^−1^. In panel (*a*), X-rays only penetrate into the surface layer as an evanescent wave with a typical penetration depth of ∼10 Å, where the image can be made to be surface sensitive. The image in panel (*b*) is taken at around the *Q*_c_ of Ti, where a weak contrast of the pattern is observed. The *Q_z_* = 0.0422 Å^−1^ is below the *Q*_c_ of the Ni layer and that of the Au layer. At this *Q_z_* the fraction of X-rays penetrating through the Ti layer (penetration depth depends on *Q_z_*) is totally reflected by the Ti/Au or Ti/Ni interfaces in the pattern regions and weakly reflected by the Ti/Si interface in the pattern-free regions. The difference in the penetrating fraction of X-rays leads to the light contrast. In panel (*c*) the fraction of X-rays passing through the surface layer increases and a higher contrast of the patterns is obtained. In addition, as the *Q_z_* is close to the *Q*_c_ of Ni, the Au patterns produce higher reflectivity than the Ni patterns (the reflection intensities of which start to decrease around the critical wavevector transfer), thus giving a contrast between the patterns of the two different materials. At pixel [55, 15] in panel (*c*), the tail structure from the main pattern is detected, and this feature can also be found in the optical image of panel (*i*) and is consistent with the features of Fig. 3 ▸(*a*). Around pixel [60, 35] in the centre of the Au polygon, a dark spot is found, and such a feature is not found in panel (*a*), which means the feature (whether it is a hole or an inclusion) is below the surface of the uniform Ti layer and above the Au polygon. It is necessary to mention that the effective image is within the inscribed circle, and some parts of the patterns at the bottom are out of the effective viewing area. In panel (*d*) the reflection intensities from the Ni patterns decrease as *Q_z_* is larger than the *Q*_c_ of Ni, while the Au patterns keep the same visibility. The visibility of the bottom-left Ni rectangle is poorest, which obviously suggests that its XR profile is different from those of the other Ni patterns, demonstrating that the layer properties (thickness or roughness) of the bottom-left Ni rectangle are different from those of the other Ni patterns. In panel (*e*) at *Q_z_* = 0.0582 Å^−1^, one can see a top-right hollow Ni triangle and a bottom-left hollow rectangle; the contrast between the edge and the centre of the patterns originates from the difference in layer thicknesses between the edge and the centre of the deposited material. The thickness difference is verified by panel (*f*) at *Q_z_* = 0.0616 Å^−1^, where the top-right Ni triangle pattern appears as a solid triangle. Besides, the bottom-left Ni rectangle shows up as an indistinct shadow and the long middle Ni bar is still highly visible, which appears to indicate that the three Ni layers have distinctive properties. In addition, the dark spot around pixel [60, 35] is still present with similar morphology. In panel (*g*) at *Q_z_* = 0.0845 Å^−1^, which is larger than the *Q*_c_ of Au, the reflection intensities from the Au patterns also begin to decline. Furthermore, the shape of the dark spot around pixel [60, 35] changes a little, revealing that the heterostructure has a depth profile inside the Au layer. At *Q_z_* = 0.0616 Å^−1^ in panel (*h*), a hollow top-left Au polygon and a hollow bottom-right Au rectangle are observed, suggesting similar thin edge structures to those of the Ni patterns. Interestingly, a long tail appears to extend from the dark spot around pixel [60, 35], which confirms that the defect stretches into the Au layer and has a depth dependence.

### 3.4. Micro-X-ray reflectivity profiles   

**Figure 5:**
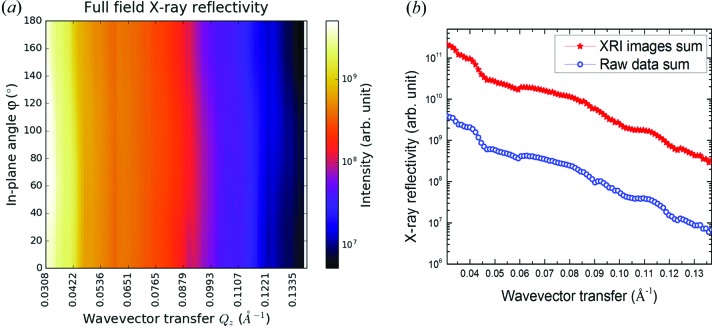
The XR profile of the whole sample. (*a*) Integrated XR of the heterogeneous thin film sample at different in-plane angles, showing the figure of merit of the measurement. (*b*) The integrated XR profiles of the sample derived from the raw data (blue open circles) and reconstructed XR images (red solid stars).

Since a series of XR images sampled equally over a range of wavevector transfers are collected, an XR profile at every micro-sized pixel can be extracted. Such an XR profile of one micro-sized pixel is called µXR. In this proof-of-principle experiment, imaging of the 90 × 90 pixels (size ∼96 µm) produces 8100 µXR profiles. Compared with nano-/microbeam (Sakurai *et al.*, 2007 ▸; Ice *et al.*, 2011 ▸; Stangl *et al.*, 2013 ▸) scan methods, although the µXR approach requires some numerical analysis, it has several merits: (i) It possesses no perspective effect based on the image reconstruction scheme; in the scan method, the size of the nano-/microbeam will be asymmetric in terms of the grazing-incidence geometry, as in the X-ray forward direction the *s* = 1 µm beam will have a footprint length of *L* = *s*/sinθ = 100 µm at θ = 10 mrad. (ii) The spatial resolution of the µXR measurement is limited by the pixel size of the area detector, although it is possible to go beyond this limitation by, for instance, employing a post-magnifier. (iii) Other than employing sophisticated optics to focus X-rays, µXR applies a parallel synchrotron beam, and this is especially important for XR which demands high angular resolution. Even so, it is important to take good care as µXR profiles are eventually calculated by an image reconstruction method. In order to check the measurement’s figures of merit, all pixel counts along the one-dimensional projection of the reflectogram (as selectively shown in Fig. 3 ▸) have been summed at each in-plane angle. The integrated profile corresponds to the XR of the heterogeneous film measured by an ordinary X-ray reflectometor. Fig. 5 ▸(*a*) shows the integrated XR profiles plotted as a function of in-plane angle. It indicates that the integrated XR profile does not change when the sample is rotated in-plane. This simple fact implies at least two important conclusions: (i) the footprint of the X-rays on the sample remained the same during the in-plane angle scan; (ii) the incidence angle did not change during the in-plane angle scan, which ensured the reproducibility of the wavevector transfer range. Fig. 5 ▸(*b*) presents a comparison of the integrated XR profile from the raw reflectogram data (blue open circles) and that from the pixel sum of µXR profiles (red solid stars). As shown, the two XR profiles are almost the same, indicating that the image reconstruction does not exhibit any preference for low- or high-reflectivity intensities.

#### 3.4.1. Micro-X-ray reflectivity profiles of arrays of pixels   

**Figure 6:**
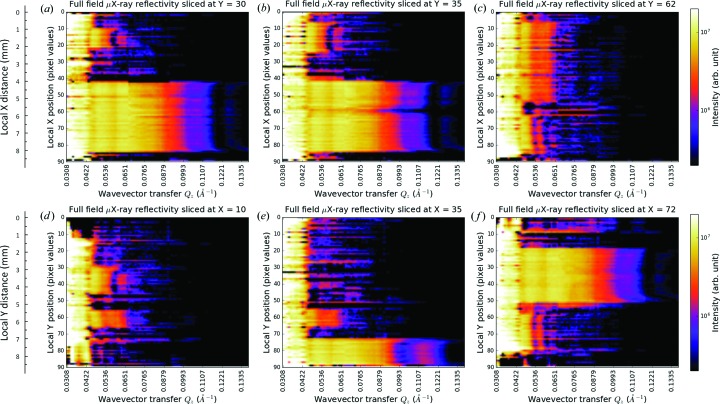
Selected µXR maps of an array of pixels at (*a*) *Y* = 30, (*b*) *Y* = 35, (*c*) *Y* = 62, (*d*) *X* = 10, (*e*) *X* = 35 and (*f*) *X* = 72, corresponding to the coordinates in Fig. 4 ▸ extracted from reconstructed XR images over the whole range of wavevector transfers *Q_z_* = 0.0308–0.1369 Å^−1^. The µXR intensity profiles are from the local (*X*, *Y*) positions indicated by the title and *y* axis of each panel, which are different from the integrated reflectograms shown in Fig. 3 ▸. The *y* axis indicates the other micro-pixel coordinate (the main micro-pixel coordinate is shown in the title of each panel), while the *x* axis corresponds to the wavevector transfer. The data are plotted on a logarithmic colour scale.

Selected µXR maps of an array of pixels are shown in Fig. 6 ▸ for (*a*) *Y* = 30, (*b*) *Y* = 35, (*c*) *Y* = 62, (*d*) *X* = 10, (*e*) *X* = 35 and (*f*) *X* = 72 (here the upper case relates to the title of each panel) for the wavevector transfer range of *Q_z_* = 0.0308–0.1369 Å^−1^, where the coordinates correspond to those of Fig. 4 ▸. The reconstructed µXR maps are pure XR profiles from an array of micro-pixels (µ-pixels), which are different from the reflectograms of Fig. 3 ▸ (a collection of data, integrated X-ray reflectograms along a perspective direction according to the Radon transform). The top panels show µXR profiles from the array of pixels along the *X* direction. In panel (*a*) at *Y* = 30, two sets of µXR profiles are seen as the *Y* = 30 line slices through the top Au polygon (*x* = 42–86, where here the lower case indicates the *y* axis of each panel) and Ni triangle (*x* = 10–20). By examining the µXR profiles with a reasonable spatial resolution, it is possible to conduct micro-area analyses of the ultrathin film sample. The centre of the Au polygon (*x* = 44–84) is quite uniform and at different locations along the *Y* = 30 line similar µXR profiles are observed, regardless of intensity fluctuations from the ring artifacts. There exists a length (∼2 pixels, 192 µm) with smaller thickness at both edges. The Ni triangle with the intercept length of 1.05 mm (*x* = 10–21, 11 pixels) is not as uniform as the Au polygon and shows an asymmetric thickness gradient at the edges (see the µXR profiles near *x* = 10 and *x* = 20). In panel (*b*) at *Y* = 35, the slice passes through the same patterns. The differences compared with panel (*a*) are as follows: a longer intercept (1.63 mm) across the Ni triangle (*x* = 7–24, 17 pixels), which is not surprising for a triangle shape; and a low-intensity profile shown at *x* = 60 in the µXR set of the Au polygon, similar to the discussion in §3.3. Moreover, the defect becomes wider at higher *Q_z_*, indicating that the defect possesses volume in a deeper location. Since the technique is an interface-sensitive imaging approach by collecting many XRI images at a series of wavevector transfers, it is a powerful method to find tiny heterostructures (usually such tiny differences control useful functions) in quite large samples. At *Y* = 62 in panel (*c*), the line goes through the bottom-left Ni rectangle and the centre-right Ni bar, and two sets of µXR profiles are seen. The two µXR profiles differ in appearance from the top-right triangle [shown in panel (*b*)]. A detailed comparison will be given in §3.4.2.

The bottom panels give µXR profiles from the array of pixels along the *Y* direction. The panel (*d*) *X* = 10 shows the local µXR profiles of the top-right Ni triangle and the centre-right Ni bar. The µXR profiles from the same pattern are similar at different perspectives, which is in agreement with the design of the sample. At *Q_z_* = 0.050–0.065 Å^−1^, the two Ni patterns obviously possess two different µXR profiles, which confirms that they differ in structure. In panel (*e*) at *X* = 35, the µXR profiles of the bottom-right Au rectangle are shown at *y* = 74–88. Compared with that of the top-left Au polygon, they have a different appearance at *Q_z_* > 0.080 Å^−1^. Furthermore, inside the Au rectangle pattern, a heterogeneous structure exists at the edges (*y* = 74–77, 3 pixels). In panel (*f*) at *X* = 72, the µXR profiles of the top-left Au polygon can be checked again. Along the *Y* direction (*X* = 72), the polygon shows an obviously asymmetric thickness gradient at the edges (compare the µXR profiles near *y* = 20 and *y* = 50). Thanks to the many local µXR profiles obtained from the measurement, more detailed analyses of the heterogeneous thin film sample can be given.

#### 3.4.2. Micro-X-ray reflectivity from single pixels   

**Figure 7:**
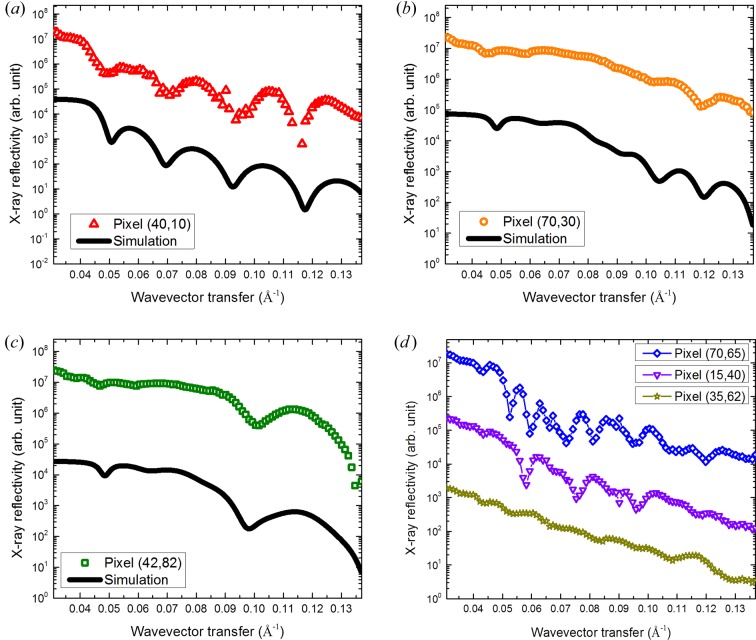
Selected µXR profiles extracted from reconstructed XR images over the whole range of wavevector transfers *Q_z_* = 0.0308–0.1369 Å^−1^ at local positions of (*a*) pixel [40, 10] (red open upward-pointing triangles), (*b*) pixel [70, 30] (orange open circles), (*c*) pixel [42, 82] (olive open rectangles), (*d*) pixel [70, 65] (blue open diamonds), pixel [15, 40] (violet open downward-pointing triangles), pixel [35, 62] (dark yellow open stars), where pixel numbers correspond to those in Fig. 4 ▸. In panels (*a*)–(*c*), simulations calculated by Parratt’s formalism are also displayed (black lines) as guides.

Fig. 7 ▸ shows several selected µXR profiles from single pixels, where the coordinates are coherent with those of Fig. 4 ▸. In panels (*a*)–(*c*), simulations calculated by Parratt’s formalism (Parratt, 1954 ▸) are displayed (black lines) as guides. The parameters used to calculate the profiles are summarized in Table 1 ▸. The pixel [40, 10] in panel (*a*) of Fig. 7 ▸ corresponds to an Au or Ni pattern-free area, which means that there is only a uniform layer of Ti at this pixel. The µXR profile confirms this point by displaying a sharp drop at *Q_z_* = 0.042 Å^−1^ and equal-period interference fringes (interference of X-rays reflected by the surface and the Ti/Si interface). The one-layer model simulation matches the profile well, irrespective of a few outliers. In panel (*b*) at the pixel [70, 30], the µXR has an intensity drop around *Q_z_* = 0.042 Å^−1^ (the *Q*_c_ of surface Ti) and shallow oscillations (due to the interference of X-rays reflected by the surface and the Ti/Au interface) below *Q_z_* = 0.08 Å^−1^ (the *Q*_c_ of Au). Beyond *Q_z_* = 0.08 Å^−1^, the µXR profile drops sharply (X-rays penetrate into the Au layer) and experiences deep oscillations (due to the interference of X-rays reflected by the surface, the Ti/Au interface and the Au/Si interface). In the simulation, it is assumed that the same properties apply to the Ti layer, and it is found that the thickness of the Au layer is around 240 Å, as shown in Table 1 ▸. The other µXR Au pattern, at the pixel [42, 82] in panel (*c*), however, shows a different oscillation period beyond *Q_z_* = 0.08 Å^−1^. Only one interference fringe is observed, which means this Au layer is thinner than that of panel (*b*). The simulation shows that the thickness of the Au layer at the pixel [42, 82] is around 112 Å, which is a reasonable value considering the difference in deposition time. Panel (*d*) gives the µXR profiles of the Ni patterns. Here no simulation has been conducted as the Ni layer is not a single layer (even in the case of uniform one-time deposition). In order to discuss the depth dependence of the structure of a multilayer, a longer *Q_z_* range and better Δ*Q_z_* resolution are necessary. Even so, it is possible to conduct such experiments for more complicated samples. In panel (*d*), µXR profiles of three pixels stand for three different Ni patterns. All three µXR profiles have a small intensity drop near *Q_z_* = 0.042 Å^−1^, which corresponds to the *Q*_c_ of Ti. The second large intensity drop in the µXR profiles corresponds to the *Q*_c_ of Ni [here *Q*_c_(Ni) = 0.049 Å^−1^]. It turns out that the Ni patterns have a low density of 5.86 g cm^−3^. Moreover, the number of interference fringes of the three µXR profiles in the limited *Q_z_* range are different: *N*_[70,65]_ > *N*_[15,40]_ > *N*_[35,62]_, indicating the difference in thicknesses *d*_[70,65]_ > *d*_[15,40]_ > *d*_[35,62]_, which is consistent with the different deposition times. In the above, µXR profiles have been successfully retrieved from the in-plane angle scan and grazing-incidence angle scan measurements.

### 3.5. Quantitative analysis and outlook   

This study has demonstrated for the first time interface-sensitive imaging by an image reconstruction aided X-ray reflectivity technique. By combining in-plane angle and grazing-incidence angle scans, µXR profiles can be extracted from the full area of a large sample. In this proof-of-principle experiment, the analysis is still semi-quantitative. Even so, it is possible to extract reliable information by applying mathematical methods like Fourier analysis (Sakurai & Iida, 1992 ▸; Voorma *et al.*, 1997 ▸). Potential future improvements include the following: (i) More careful calibrations of the direct beam intensities and the detector. In order to extract XR profiles to analyse a film’s properties like roughness, it is necessary to apply normalization to the XR projections. Moreover, it is important to consider the sensitivity of the area detector to different intensities, since XR covers quite a large dynamic range. (ii) The use of a robust image reconstruction scheme. It is worth considering introducing some suitable image reconstruction approaches to obtain reliable numbers in the inverse processes. Sometimes regularizations are required, and then it is necessary to know the resolution matrix to see how the results are smeared out.

## 4. Conclusion   

In conclusion, interface-sensitive imaging of a heterogeneous thin film sample by an image reconstruction aided X-ray reflectivity technique has been successfully demonstrated employing a wide monochromatic synchrotron beam. By applying an area detector, and combining in-plane angle and grazing-incidence angle scans, a series of XR images at different grazing-incidence angles (proportional to wavevector transfers) are obtained by mathematical image reconstruction. The physical meaning of a reconstructed XR image at a specific wavevector transfer is the two-dimensional reflectivity distribution of the sample. It has become possible to collect the µXR (where the pixel size is on the microscale) profiles at different local positions of the sample, where the spatial resolution of the µXR measurement is decided by the pixel size of the reconstructed XRI images.

## Supplementary Material

Supplementary Video 1 demonstrates the full collection of the X-ray reflectivity sinograms of the sample at different grazing angles (corresponding to different wavevector transfers Qz). The difference of wavevector transfers between consecutive frames is Delta Qz = 0.00114 Å−1. The different frames are plotted on a same range logarithmic colour scale for comparison. In each frame, the sinograms are plotted as a function of in-plane angle phi, where the scanning step for the in-plane angle is Delta phi = 2 deg. Video 1 displays how the X-ray reflectivity sinogram pattern (in-plane structure) changes with different Qz or different X-ray depth-dependent intensity profiles in the sample.. DOI: 10.1107/S160057671700509X/vc5005sup1.wmv

Supplementary Video 2 covers the full collection of the X-ray reflectograms of the sample at different in-plane angles. The difference of in-plane angles between consecutive frames is Delta phi = 2 deg. The different frames are plotted on a same range logarithmic colour scale for comparison. In each frame, the reflectograms are plotted as a function of wavevector transfer Qz, where the scanning step for the wavevector transfer is Delta Qz = 0.00114 Å−1. Video 2 exhibits how the X-ray reflectogram pattern (X-ray reflectivity profile integrated along the X-ray forward direction) changes with different in-plane angle or different perspective.. DOI: 10.1107/S160057671700509X/vc5005sup2.wmv

## Figures and Tables

**Table 1:** The parameters used for Parratt’s formalism to simulate the XR profiles in Fig. 7 ▸ The inter-diffusion parameter is set as 16 Å for the Ti surface, 10 Å for the Ti/Au interface and 6 Å for the Au/Si interface. Local points correspond to the positions specified by the pixel values in Fig. 4 ▸. The silicon substrate has an infinite thickness. The layer density ρ is calculated from the *Q*_c_ and is that of the layer indicated in bold.

Local points	Layers model	Measured *Q*_c_ (Å^−1^)		Layer density (g cm^−3^)	Thickness (Å)
[40, 10]	**Ti**/Si	0.04108		4.25	230
[70, 30]	Ti/**Au**/Si	0.07936		19.32	230/280
[42, 82]	Ti/**Au**/Si	0.07936		19.32	230/112
